# The Regulatory Role of Quorum Sensing-Mediated Amino Acid Metabolism in Biofilm Formation and Motility of *Hafnia alvei* H4

**DOI:** 10.3390/foods15020281

**Published:** 2026-01-12

**Authors:** Congyang Yan, Xue Li, Gongliang Zhang, Jingran Bi, Hongshun Hao, Hongman Hou

**Affiliations:** 1 School of Food Science and Technology, Dalian Polytechnic University, Dalian 116000, China; yancy1987@outlook.com (C.Y.); lixuexiaomuzhu@163.com (X.L.); zhanggl1978@hotmail.com (G.Z.); bijingran1225@foxmail.com (J.B.); 2 Liaoning Key Lab for Aquatic Processing Quality and Safety, Dalian 116000, China; beike1952@163.com; 3 College of Grain Science and Technology, Shenyang Normal University, Shenyang 110034, China

**Keywords:** *Hafnia alvei*, quorum sensing, bacterial motility, bacterial biofilm, serine

## Abstract

The spoilage phenotype of microorganisms is a key mechanism leading to food spoilage, but how their metabolic environment affects the spoilage phenotype remains unclear. This study utilized metabolomics and spoilage phenotype analysis to reveal metabolic differences between different quorum sensing (QS) genotypes of *Hafnia. alvei* H4 and their impact on spoilage phenotypes. Ultra-high performance liquid chromatography–fluorescence detection revealed that the QS system participated in the differential metabolic regulation of eight amino acids, with serine exerting the most significant influence on the spoilage phenotype. Subsequent studies demonstrated that QS-promoted serine inhibited bacterial motility by affecting the biosynthesis of rhamnolipid (rather than c-di-GMP) and inhibiting flagellar/chemotactic genes expression. Moreover, QS-promoted serine induced the difference of bacterial inner membrane, further inhibiting bacterial motility. These findings provided fundamental information for the control of biofilms conformation within complex food nutritional background.

## 1. Introduction

Food spoilage is frequently caused by the colonization and proliferation of spoilage microorganisms. Nutrient-rich and high-value foods, such as meat and fish, are often more prone to spoilage than other foods, which affect human health and causes huge economic costs [1]. *Hafnia alvei* is a widespread foodborne spoilage and pathogenic bacteria, widely isolated from a variety of foodstuffs, including meat, ready-to-eat foods and seafoods [2]. Due to these characteristics, *H. alvei* often causes serious nutritional loss and safety problems in food industry by producing extracellular enzymes [3], spoilage metabolites [4], and forming biofilms [5]. Recent studies have revealed that the regulation of spoilage phenotype is closely associated with a density-dependent regulatory mechanism known as the quorum sensing (QS) system [6,7].

As one of the important regulatory mechanisms, QS rely on specific diffusible chemical signals to coordinate various group behaviors, including nutrient metabolism, biofilm formation, and bacterial motility [8,9,10]. Biofilms are highly organized microcolony aggregates formed by bacteria adhering to food surfaces, enveloped by an extracellular polymeric matrix secreted by the bacteria themselves [11], so biofilms mainly increase the survival rate of spoilage bacteria in food processing by enhancing bacterial adhesion and resistance. It is widely recognized that the formation of biofilms relies on membrane proteins, genes products of the cytotoxin-associated gene pathogenicity island, which express a type IV secretion system (cag-T4SS), and flagella genes. Flagellar filaments have been shown to be part of the biofilm matrix along with secreted proteins, eDNA, carbohydrates, and LPS [11]. The transition between bacterial motility and biofilm formation represents a fundamental lifestyle switch, where motility facilitates surface colonization and biofilm initiation, while subsequent adhesion and matrix production stabilize the sessile community. This dynamic process is critically regulated by several key factors. Surface-active compounds like rhamnolipids not only promote swarming motility by reducing surface tension but also contribute to biofilm architecture and later dispersal [12]. Concurrently, the intracellular second messenger cyclic di-GMP (c-di-GMP) functions as a central signaling hub, with high levels typically promoting biofilm formation and repressing motility, whereas low levels favor the motile state [13]. Therefore, the food substrate and metabolic environment in which spoilage bacteria survive were also important factors determining the process of biofilm formation. To date, many studies have examined the effects of QS system components on biofilm formation [14,15], while few have explored the role of QS-regulated metabolites on biofilm formation.

Our previous research systematically investigated the QS system of *H. alvei* H4, elucidated the growth-phase-dependent regulatory role of QS on metabolic pathways, biofilm formation and swimming motility [6,7]. These foundational works established a clear correlative link between QS functionality and key spoilage phenotypes in this specific strain. However, it remains unknown how the QS signal is translated into phenotypic changes through the regulation of specific metabolites. In this study, a regulatory network of QS-regulated metabolites affecting bacterial biofilm and motility was constructed. QS-regulated metabolites were screened and identified by untargeted metabolomics and UHPLC-FLD, respectively. Subsequently, the effects of these differentially metabolites on disrupting bacterial biofilm formation and motility were evaluated. Based on the key metabolites affecting cell motility (rhamnolipids and c-di-GMP) and motility-related functional building blocks, the regulation mechanism of QS-regulated metabolites affecting cell motility was preliminarily studied. This study preliminarily explored the underlying mechanism by which QS affects spoilage phenotypes through the regulation of key metabolites, providing a basis for more precise regulation of quorum sensing-induced spoilage phenotypes.

## 2. Materials and Methods

### 2.1. Bacterial Strains and Culture Conditions

*H. alvei* H4 wild-type strain (WT, CCTCC AB 2019337) and its isogenic QS mutants (Δ*luxI*, Δ*luxR* and Δ*luxIR*) were cultured overnight in Luria–Bertani (LB) unless noted. Strains were inoculated into assay medium at an optical density of 0.05 at 600 nm (OD_600_) and incubated at 30 °C.

### 2.2. Motility Assays

These strains overnight culture were washed with physiological saline and used to inoculate the assay medium. Swimming motility assay were performed according to the method described by Vivas et al. [16]. 4 µL of culture was spotted on swimming agar medium, which contained 1% tryptone, 0.25% NaCl and 0.3% agar. The swimming diameter was photographed after 60 h at 30 °C. To observe the swarming motility, the bacterial colonies were inoculated onto the swarming agar medium contained 1% tryptone, 0.25% NaCl, and 0.6% agar. After incubation at 30 °C for 60 h, the swarming diameter was measured. In motility assays comparing different QS genotype strains, the WT strain served as the control, whereas in the experiments investigating the influence of QS-regulated amino acids on swimming ability, the control was defined as the group without exogenous amino acid supplementation.

### 2.3. Colony Biofilm Assay

The protocol for this assay was adapted from Ausubel et al. [17]. Briefly, aliquots of *H. alvei* WT and its isogenic QS mutants were washed twice with phosphate-buffered saline (PBS) and spotted onto 60 × 15 mm Petri plates containing 25 mL of 0.5% yeast extract, 1% tryptone, 1.5% agar, 0.004% Congo red dye, and 0.002% Coomassie brilliant blue dye. Colonies were grown at 30 ˚C and images were acquired after 6 days.

### 2.4. Extraction and Quantification of c-di-GMP

C-di-GMP was extracted as described previously [17]. Targeted strains were cultivated in 100 mL of LB or swimming medium at 30 °C. Cells were harvested at 12 h by centrifugation at 8000× *g* for 10 min. The bacteria were washed with 40 mL of PBS containing 0.18% formaldehyde and centrifuged at 8000× *g* for 10 min at 4 °C. The precipitate was boiled in water for 12 min, then cooled on ice for 10 min, after which nucleotides were extracted with 65% ethanol. The extraction process was repeated on the precipitate. The collected supernatant was freeze-dried and redissolved in 1 mL of 0.15 M triethylammonium acetate. The sample was filtered through a 0.22 μm polyvinylidene fluoride (PVDF) membrane and analyzed by high-performance liquid chromatography (HPLC) using a reversed-phase column. For the comparison of c-di-GMP levels among different QS genotype strains, the WT strain was used as the control to examine the regulatory role of the QS system on c-di-GMP. In experiments investigating the effects of exogenous serine supplementation at varying concentrations on c-di-GMP, the group without serine addition served as the control.

### 2.5. Rhamnolipid Analysis

The rhamnolipid production in the various bacterial cultures was quantified as described previously [18]. The overnight cultured was centrifuged at 8000× *g* for 10 min to remove the bacteria. 300 µL of the supernatant was extracted twice with 700 µL diethyl ether, followed by freeze-drying of the ether fraction. The resulting residue was redissolved in 100 µL of distilled water, then mixed with 800 µL of 60% sulfuric acid and 100 µL of 1.6% orcinol. The mixture was heated at 80 °C for 35 min. the OD_421_ value of samples were measured. Based on the assumption that 1 µg of rhamnose corresponds to 2.5 µg of rhamnolipid, the content of rhamnose in the sample was determined [19]. For the comparison of rhamnolipid production among different QS genotype strains, the WT strain served as the control. In experiments investigating the influence of exogenous serine supplementation at varying concentrations on rhamnolipid metabolism, the group without serine addition was designated as the control.

### 2.6. Conditioned Medium from H. alvei WT and Its Isogenic QS Mutants

The preparation of conditioned medium (CM) was obtained as described by Albuquerque et al. [20]. The CM of targeted strains was generated by growing cultures with an initial OD_600_ 0.05 in LB for 12 h, removing the cells by centrifugation at 8000× *g* for 10 min.

### 2.7. Motility in the Presence of CM

*H. alvei* WT cells overnight in LB at 30 °C were collected by centrifugation, washed three times with PBS (pH 7.2), and spotted onto swimming medium complemented with different concentrations of CM. The swimming diameter was photographed after 60 h at 30 °C. The control was the group supplemented with CM prepared from the WT strain.

### 2.8. Metabolite Measurements of CM

The WT and Δ*luxI* was cultured overnight in LB medium, and then were collected and centrifuged at 15,000 rpm for 5 min. The supernatants were harvested and quickly stored at −80 °C prior to use. Various metabolites were determined according to the instructions of the manufacturer.

### 2.9. Amino Acid Analysis

Amino acid analysis was performed as described by Tuberoso et al. [21]. The *H. alvei* WT and Δ*luxI* were inoculated to an OD_600nm_ 0.15 in LB and grown for 12 h. 1 mL of media was collected and pelleted for 10 min at 8000 rpm. Supernatant was derivatized with Dansyl chloride (DCl), which consisted of 70 µL of sample, 100 µL of DCI and 0.2 M Na_2_B_4_O_7_-10H_2_O (pH 9.3) solution up to a final volume of 1000 µL. The mixture was incubated for 30 min at 40 °C in an ultrasonic bath, and then centrifuged at 12,000 rpm for 10 min. The supernatant was recovered and diluted with MeOH (1:1, *v*/*v*) for ultra-performance liquid chromatographic analysis-fluorescence detection (UHPLC-FLD) analysis. Using the WT strain as a control to demonstrate the regulatory role of the QS system on amino acid metabolism.

### 2.10. Cystal Violet Biofilm Assays

Biofilm assays were performed as previously described by Yan et al. [22]. The overnight bacteria were diluted to a final OD_600nm_ 0.05 in LB broth supplemented with different concentrations of serine. Aliquots (2 mL) were transferred to a 15 mm glass tube and incubated at 37 °C for 24 h. After removing the culture medium, glass tubes were washed twice with PBS (pH 7.2) and the attached bacteria stained with 0.1% (*v*/*v*) crystal violet for 15 min. The cell pellet was then washed and rinsed with PBS (pH 7.2) until all unbound dye was removed. The crystal violet was solubilized by 33% glacial acetic acid and biofilm amount estimated by measuring the OD_595nm_. The control was the experimental group without serine supplementation, which was used to compare the effects of adding different concentrations of serine on bacterial biofilm formation.

### 2.11. Observation of Biofilm by Fluorescence Microscopy

Biofilm assays in coated 6-well polystyrene plates were performed as previous study [23]. In brief, *H. alvei* H4 was cultured in LB media supplemented with various concentrations of serine. The wells after removing the supernatant were gently washed twice with distilled water. The SYTO 9 was added to each well with equal volumes of propidium iodide, and then the well was incubated in the dark for 15 min. The dye mixture was discarded and the stained images of biofilms were acquired by using Nikon Eclipse Ti inverted fluorescence microscope (Nikon, Tokyo, Japan).

### 2.12. Quantitative Real-Time RT-PCR

Total RNA samples were prepared from swimming medium with or without 5 mM or 40 mM serine using RNAprep pure Cell/Bacteria Kit (TIANGEN, Beijing, China). cDNA was synthesized from 1000 ng RNA using a PrimeScript^TM^ Reagent kit (Takara, Dalian, China). Amplification was performed with a Step-One Thermal Cycler (Applied Biosystems, Foster City, CA, USA). *16s rRNA* was used as a housekeeping control. The abundance of mRNA was calculated using the comparative 2^−ΔΔCt^ method. The control was the experimental group without serine supplementation, which was used to compare the effects of adding different concentrations of serine on the expression of genes related to rhamnolipid, c-di-GMP, flagella, chemotaxis, electron transfer, and QS.

### 2.13. Measurement of Membrane Potential

Membrane potential was measured according to a previous study [24]. Cells were grown in swimming agar medium supplemented with 10 μM ThT, a cationic dye that can accumulate in cells due to their inside-negative electrical membrane potential (the dye accumulation thus negatively correlates to the cell membrane potential), so the fluorescence intensity in the cells therefore increases. Cells were then collected from the above medium and observed by fluorescence microscope (em/ex: 450/482 nm).

### 2.14. Statistical Analysis

Statistical analyses were performed using GraphPad Prism (version 8.3.0). In all assays, data were expressed as mean ± standard deviations (SD). Statistical significance was determined using one-way ANOVA or two-tailed *t* test. *p* values were reported using the following symbolic representation: NS (No significance) *p* > 0.05; * *p* < 0.05; ** *p* < 0.01; *** *p* < 0.001; **** *p* < 0.0001.

## 3. Results

### 3.1. Phenotypic Analysis of H. alvei WT and Its Isogenic QS Mutants in Different Cultures

**Figure 1:**
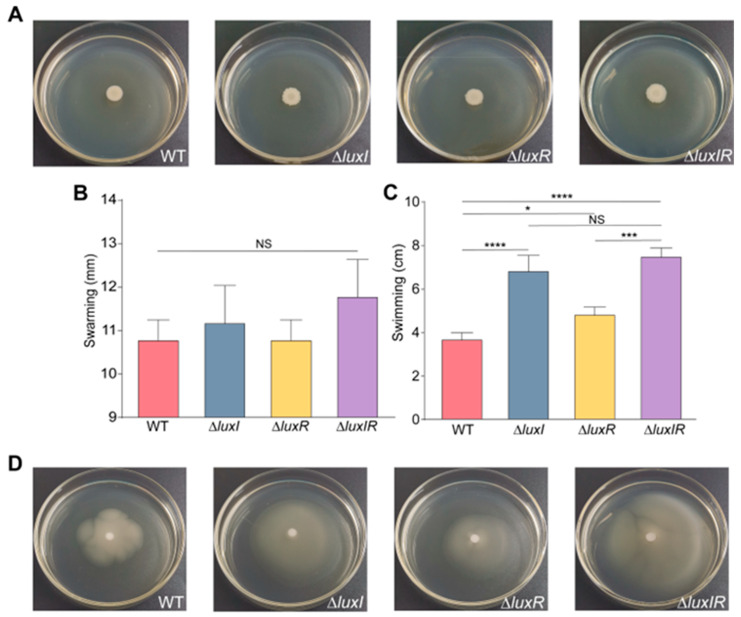
Phenotypic analysis of various QS genotype strains of *H. alvei* H4. Swarming phenotype (**A**) and colony diameter statistics (**B**) of *H. alvei* H4 WT and its isogenic QS mutants at 30 °C after 60 h. Swimming colony diameter statistics (**C**) and phenotype (**D**) of above strains after 60 h of incubation at 30 °C. Data are averages of two independent experiments (*n* = 5). Error bars represent the standard deviation (SD) of biological replicates. Statistical comparisons were performed using one-way ANOVA of GraphPad software. * *p* < 0.05; *** *p* < 0.001; **** *p* < 0.0001. NS means not significant.

In order to investigate the effects of QS genes and nutritional metabolites on bacterial phenotype, the phenotypes of *H. alvei* WT and its isogenic QS mutants were analyzed under different cultural backgrounds. In the first series of experiments, although WT and its isogenic QS mutants swarmed on the surface of swarm plates at 60 h, the isogenic QS mutants abolished swarming like the WT strain (Figure 1A,B). However, all the isogenic QS mutants upregulated the swimming motility relative to the WT strain (Figure 1C,D).

### 3.2. Phenotypic and Physicochemical Analyses of Various QS Genotype Strains of H. alvei H4

**Figure 2:**
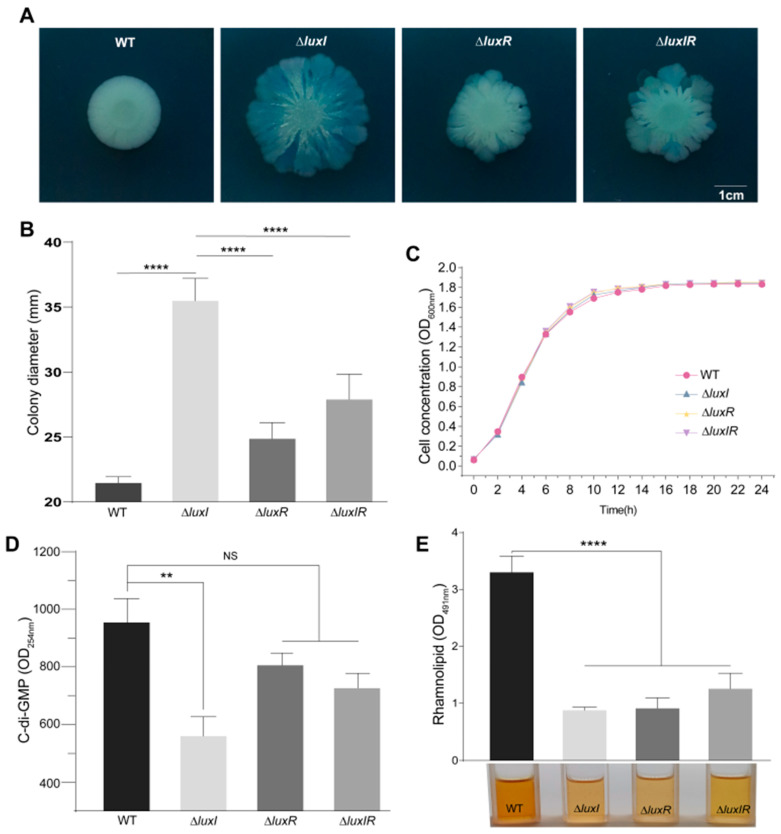
Phenotypic and physicochemical analyses of various QS genotype strains of *H. alvei* H4. Colony phenotype (**A**) and colony diameter (**B**) of *H. alvei* H4 WT and its isogenic QS mutants on Congo-red plates after 6 d at 30 °C. Growth curves (**C**), c-di-GMP (**D**), and rhamnolipids (**E**) of these strains. Data are averages of two independent experiments (*n* = 5). Error bars represent the standard deviation (SD) of biological replicates. Statistical comparisons were performed using one-way ANOVA of GraphPad software. ** *p* < 0.01; **** *p* < 0.0001. NS means not significant.

For Congo-red phenotype, we found that the isogenic QS mutants formed a wrinkly colony when it was grown on Congo-red plates at 30 °C for 6 d, while the WT strain formed white smooth colonies (Figure 2A), and found that the colony diameter of the Δ*luxI* strain increased by 167% compared with the WT strain (Figure 2B). These results showed that bacteria with different QS genotype exhibited the same phenotype under the same culture conditions (swarming phenotype), and bacteria with the same QS genotype exhibited different phenotypes under different culture conditions, indicating that nutritional environment play a role in the regulation of colony phenotype. Motility, an important factor affecting the above phenotypes, was negatively and positively influenced by cyclic diguanylate (c-di-GMP) and rhamnolipids, respectively [17]. We subsequently ruled out the effect of biomass on target metabolite differences by measuring the growth of *H. alvei* WT and its isogenic QS mutants (Figure 2C), and found that the content of c-di-GMP (Figure 2D) and rhamnolipids (Figure 2E) in the supernatant of the WT strain was significantly higher than that of the isogenic QS mutant strain. This showed that different QS genotypes of *H. alvei* H4 may indeed affect the colony phenotype by changing the metabolic environment, but this still needed further verification.

### 3.3. Effects of CM on Swimming Phenotype of H. alvei WT

**Figure 3:**
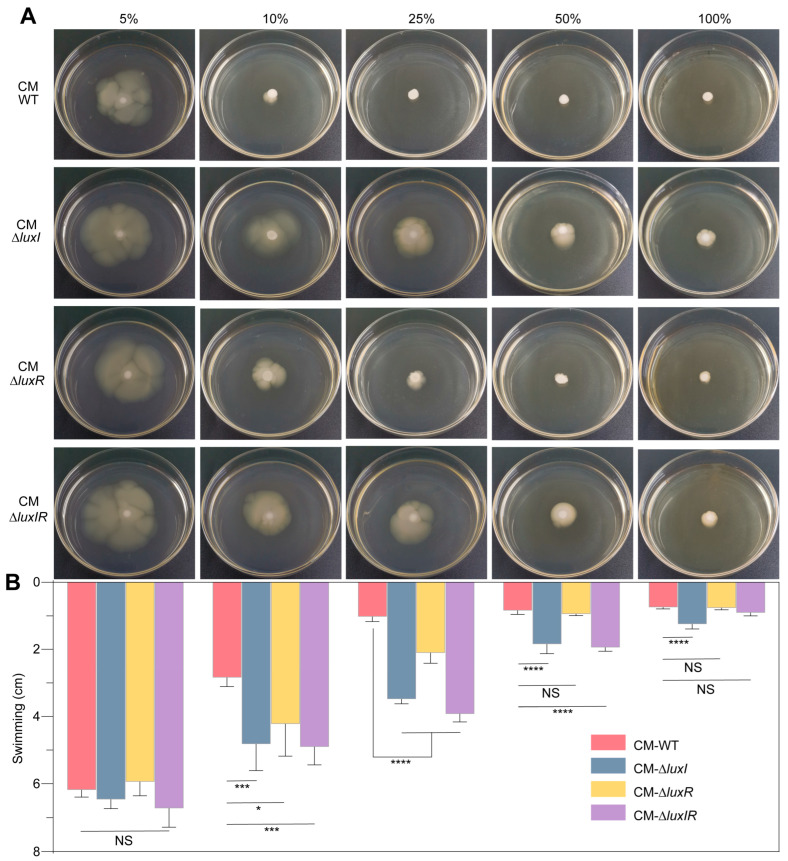
Comparison of the effects of CM obtained from *H. alvei* WT and its isogenic QS mutants on swimming phenotype. *H. alvei* WT cells were grown overnight and washed three times with PBS, and 4 μL of washed culture were spotted on swimming medium supplemented with increasing concentrations of CM obtained from *H. alvei* H4 WT, Δ*luxI*, Δ*luxR* and Δ*luxIR* (**A**). The colony diameter of each group was recorded (**B**). We can observe that CM obtained from *H. alvei* WT and Δ*luxI* causes the most significant phenotypic difference. Data represent the mean values ± SD (with five biological replicates) and were analyzed using one-way ANOVA. Values marked with asterisks are statistically significant. * *p* < 0.05; *** *p* < 0.001; **** *p* < 0.0001. NS means not significant.

To further confirm the effect of QS-regulated metabolites on colony phenotype of *H. alvei* WT, we prepared conditioned medium (CM) from cell-free supernatants of stationary-phase cultures of *H. alvei* WT, Δ*luxI*, Δ*luxR*, and Δ*luxIR* in LB medium, and then added these CM into the fresh swimming medium according to the proportion shown in Figure 3A to evaluate their effect on the swimming phenotype of *H. alvei* WT (Figure 3A). The addition of 10% CM from *H. alvei* WT or 25% CM from Δ*luxR* produced a significant inhibitory effect on the swimming phenotype, while the effect of CM from Δ*luxI* or Δ*luxIR* on the swimming phenotype was dose dependent (Figure 3B). This experiment determined that *H. alvei* WT and its isogenic QS mutants produced differential metabolites capable of interfering with colony phenotypes.

### 3.4. Metabolic Analysis of CM from H. alvei WT and ΔluxI

**Figure 4:**
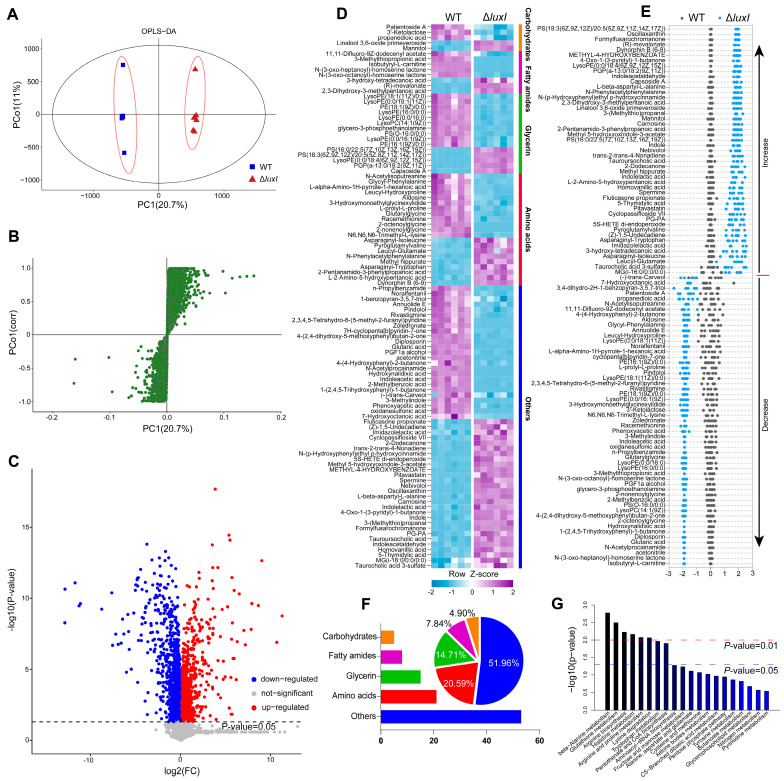
Metabolic profile analysis of CM obtained from *H. alvei* H4 WT and Δ*luxI*. OPLS-DA plots with the scores of the first two principal components (**A**). S-plots of OPLS-DA model from the WT and Δ*luxI* (**B**). Volcano plots of differential metabolite of CM from each strain (**C**). Heat map showing relative abundance of 102 significantly differential metabolites of CM obtained from WT and Δ*luxI*. Heat map scale (blue to purple, low to high abundance) is shown below data (*n* = 6) (**D**). Cluster Statistics of differential metabolites (**E**). Z-scores (standard deviation from average) corresponding to data in (**D**). Results of cluster analysis (**F**). Metabolic pathway enrichment analysis of differential metabolites (**G**).

Based on the effect of the CM obtained from different QS genotypes of *H. alvei* H4 on swimming phenotype, the phenotypic effects between CM obtained from *H. alvei* WT and Δ*luxI* was found to have the most significant differences. Therefore, these strains were selected to analyze the different metabolites in their CM that may affect the swimming phenotype. UPLC–MS was used to identify the metabolite profiles in both positive and negative ion modes and 4962 metabolites were revealed in their CM. Score plots of OPLS-DA showed that *H. alvei* WT could be separated from Δ*luxI* (Figure 4A). The fitness of the OPLS-DA model was cross-validated by using permutation analysis (Supplementary Figure S1). 474 differential metabolites were selected based on S-Plot (Figure 4B, *p* < 0.05, adjusted FDR), among which, 233 metabolites were up-regulated and 241 metabolites were down-regulated by QS (Figure 4C). The metabolites with the top 102 differences between CM obtained from *H. alvei* WT and Δ*luxI* were selected for further analysis (*p* < 0.01; Figure 4D). Of the 102 metabolites, 55 were present at lower abundance in the CM from Δ*luxI* and 47 were present at higher abundance (Figure 4E). Cluster analysis of these differential metabolites revealed that except for 51.96% of metabolites that were not assigned to a specific metabolic category, amino acids had the highest abundance of differential metabolites, reaching 20.59% (Figure 4F), which was consistent with the metabolic pathway enrichment analysis (Figure 4G). Therefore, we speculated that differential amino acids between CM obtained from *H. alvei* WT and Δ*luxI* may contribute to the generation of differential phenotypes.

### 3.5. Identification of QS-Induced Amino Acids and Their Effects on Swimming Phenotype

**Figure 5:**
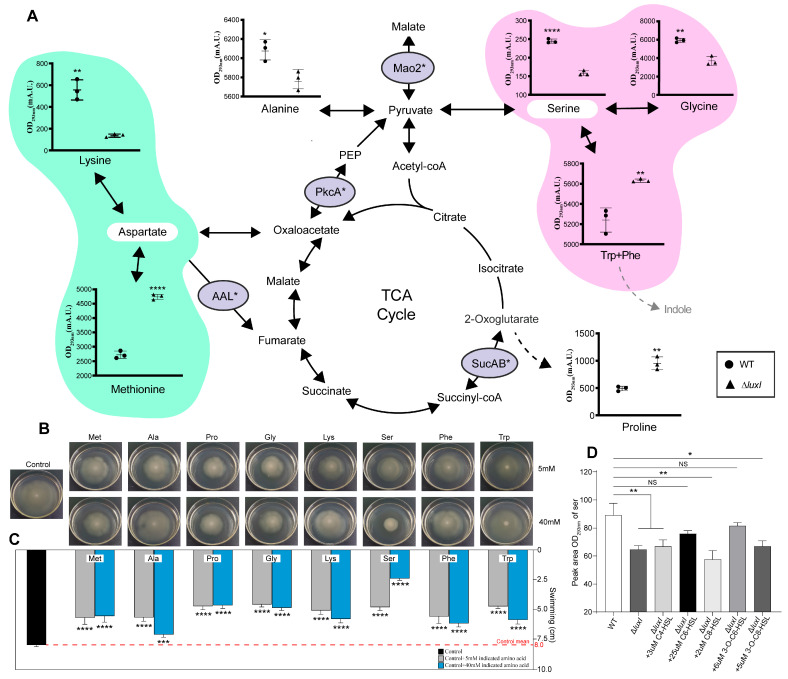
Differential amino acid analysis between *H. alvei* H4 WT and Δ*luxI* and their effects on swimming phenotypes. The pathways of amino acids that are associated with the TCA cycle were determined from the information provided in the Kyoto Encyclopedia of Genes and Genomes (KEGG). The concentration of amino acids within the CM obtained from WT and Δ*luxI* were quantitated as described in Section 2 (**A**). Data represent the mean values ± SD (three biological replicates). Statistics were achieved by a two-tailed *t* test: * *p* < 0.05; ** *p* < 0.01; **** *p* < 0.0001. Swimming phenotype influenced by differential amino acids between CM from *H. alvei* WT and Δ*luxI* (**B**) and its colony diameter (**C**). Metabolic levels of ser in the group of WT strain and Δ*luxI* with or without various exogenous AHLs (**D**). Error bars in (**C**,**D**) represent the SD of the means (*n* = 5). The data were analyzed using the one-way ANOVA: * *p* < 0.05; ** *p* < 0.01; *** *p* < 0.001 and **** *p* < 0.0001. NS means not significant.

Amino acids were quantified from both CM obtained from *H. alvei* WT and Δ*luxI* grown in LB medium. In this case, there was no significant difference in the concentrations of most amino acids between these CM, with the exception of eight amino acids, including glycine, serine, alanine, lysine, methionine, proline, phenylalanine and tryptophan (Figure 5A). Serine and methionine are known to be key intermediates in the biosynthesis of other vital metabolites. Interestingly, our study found that these two amino acids exhibited the largest differences between CM obtained from *H. alvei* WT and Δ*luxI*. These results suggested that the phenotypic differences influenced by the both CM may be due to the intervention of these amino acids. To verify this hypothesis, eight different amino acids at 5 mM and 40 mM were added to the swimming media to test whether these amino acids affected the swimming ability of *H. alvei* WT. As shown in Figure 5B, the swimming ability of *H. alvei* WT seemed to be more or less inhibited with the addition of amino acids and showed phenotypic suppression in an amino acid concentration-dependent (Met and Ser) or -independent (other amino acids) manner. Quantitative results of the above swimming phenotype showed that swimming ability of *H. alvei* WT was inhibited approximately 1.82-fold, 1.72-fold and 3.33-fold in the presence of 40 mM Pro, 40 mM Gly and 40 mM Ser, respectively, compared to that of control (Figure 5C). The above results indicated that serine had the strongest ability to inhibit the swimming ability of *H. alvei* WT. In particular, it was noted that serine was more abundant in CM obtained from *H. alvei* WT, which may be one of the factors that enable CM from WT strain to significantly inhibit bacterial motility. In order to further prove the regulatory effect of QS on serine metabolism, the AHLs addback study was carried out and the results showed that the metabolic levels of serine in the group of adding C6-HSL and 3-O-C6-HSL were returned to the level of WT strains, indicating that serine was indeed regulated by QS system (Figure 5D). In conclusion, amino acids have regulatory activity on the swimming ability of *H. alvei* WT.

### 3.6. Effects of QS-Regulated Serine on Rhamnolipids and c-di-GMP

**Figure 6:**
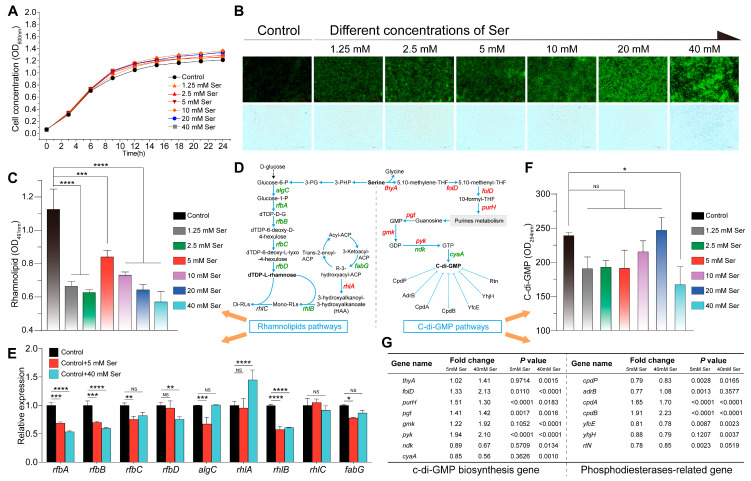
Effects of QS-regulated serine on biomass, cell aggregation, rhamnolipids and c-di-GMP. Different concentrations of serine appeared to have no significant effect on biomass (**A**). Top-down view of cell aggregation stained with SYTO 9 and propidium iodide (**B**). The effect of serine on the biosynthesis of rhamnolipid in *H. alvei* H4 in a dose-dependent manner (**C**). Metabolic pathway from serine to rhamnolipid and c-di-GMP (**D**), in which different colors represent the regulatory effect of serine on the gene expression: red, upregulation; Green, down-regulation. Regulation of serine on genes in rhamnolipid synthesis pathway (**E**). Effects of different concentrations of serine on the biosynthesis of the c-di-GMP (**F**) and its metabolic pathway genes (**G**). Data represent the mean values ± SD (three biological replicates). Statistics were achieved by one-way ANOVA: * *p* < 0.05; ** *p* < 0.01; *** *p* < 0.001; **** *p* < 0.0001; NS means not significant.

Previous results showed that the addition of exogenous serine to the swimming media exerted an inhibitory effect on the swimming ability of *H. alvei* WT. To better understand the mechanism by which serine affect swimming ability of *H. alvei* WT, we analyzed the effect of different concentrations of serine on two key metabolites related to swimming ability, rhamnolipids and c-di-GMP. First, growth monitoring ruled out the possibility that different concentrations of serine might bring about changes in the accumulation of two key factors through differences in biomass (Figure 6A). Second, the results of the fluorescence microscope showed that the degree of bacterial aggregation was positively correlated with the concentration of serine (Figure 6B), which was consistent with the results of the swimming experiment described above, implying that a certain concentration of serine may cause bacterial aggregation by inhibiting the swimming ability of bacteria. This suggested that serine may be involved in the metabolic regulation of these two key substances affecting bacterial swimming ability. To test this idea, the content of rhamnolipids in the swimming medium supplemented with different concentrations of serine was first determined (Figure 6C). As predicted by the hypothesis, it was found that rhamnolipids levels decrease in the presence of different concentrations of serine, compared to the control without adding serine. Notably, the addition of 1.25 mM serine was sufficient to significantly suppress rhamnolipid accumulation. Furthermore, serine was involved in the biosynthesis of rhamnolipids through the Glucose-6-phosphate pathway [25], which contained nine key genes discovered through the genome of *H. alvei* H4 (Figure 6D). We then investigated how serine affected rhamnolipid formation by performing quantitative reverse transcription-PCR (qRT-PCR) assays on *H. alvei* H4 in the swimming medium with or without the addition of indicated concentrations of serine. The results showed that only the *rhlA* gene was upregulated after the addition of serine, and the other seven genes were significantly down-regulated (Figure 6E), which may explain to some extent why serine could inhibit rhamnolipid production.

Next, we investigated the effect of different concentrations of serine on c-di-GMP accumulation and on the expression of selected genes involved in the pathway of serine-to-c-di-GMP. Overall, the addition of different concentrations of serine had no significant impact on the metabolism of c-di-GMP compared to the observations made from control, with only 40 mM serine significantly reducing the level of the c-di-GMP (Figure 6F), implying that serine may affect bacterial motility more by rhamnolipids than by c-di-GMP. Although the transcript levels of almost all related genes involved in serine-to-c-di-GMP pathways were upregulated by 5 mM or 40 mM serine, it is worth noting that *cyaA* encoding diguanylate cyclises (DGCs) was significantly inhibited by 40 mM serine, which was one of the factors limiting the biosynthesis of c-di-GMP by serine (Figure 6G). In addition, we found that the addition of serine significantly upregulated the transcriptional levels of *cpdA* and *cpdB* encoding 3,5-cyclic AMP phosphodiesterase and 2,3-cyclic nucleotide 2-phosphodiesterase, respectively, which belong to phosphodiesterase (PDEs) involved in the degradation of c-di-GMP. This further contributes to the inhibitory effect of serine on the accumulation of c-di-GMP. Upregulation of the activity of PDEs related genes by serine may be detrimental to the formation of biofilms. Crystal violet assays with different concentrations of serine showed that serine almost reduced biofilm formation in a dose-dependent manner (Supplementary Figure S2). Taken together, these data provided evidence that nutritional differences triggered by metabolic regulation of the microbial QS system can have a significant impact on bacterial phenotype.

### 3.7. QS Downregulated Bacterial Flagella/Chemotaxis-Related Genes and Upregulated Membrane Potential by Regulating Serine

**Figure 7:**
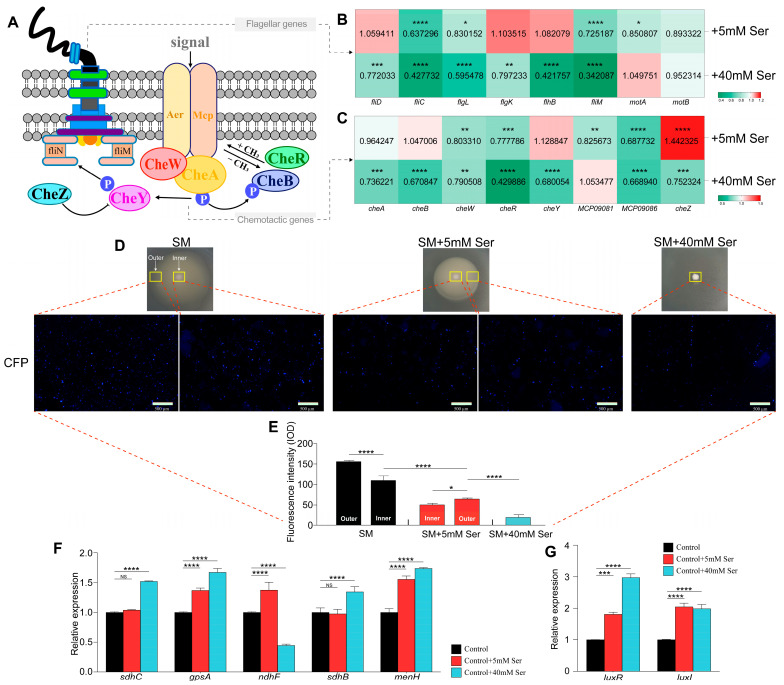
Effects of different concentrations of serine on motility-related functional building blocks in *H. alvei* H4. Schematic representation of the central motility apparatus (**A**). Heat map of the regulation of Serine on flagellum (**B**) and chemotaxis (**C**) related genes. According to 2^−ΔΔCT^ method, expression level of genes in the treatment group (adding 5 mM or 40 mM serine) against the control group (without adding serine) was calculated: >1, upregulation; <1, downregulation. The expression value of each gene in the control group is 1. Detection of the membrane potential in cells collected from the inner region of (indicated by the yellow square) a swimming agar medium adding different concentrations of serine by using the fluorescent dye thioflavin T (ThT, shown as cyan fluorescent protein (CFP)) (**D**), and the fluorescence intensity was quantified using Image Pro Plus 6.0 (**E**). Real-time PCR analyses to probe expression of the genes involved in electron transfer (**F**). Effect of Serine on QS-circuit genes (**G**). Data in (**B**,**C**,**F**,**G**) are averages of three biological replicates, while data in E are averages of five biological replicates. Error bars represent the SD of the means. The data were analyzed using the one-way ANOVA. * *p* < 0.05; ** *p* < 0.01; *** *p* < 0.001; **** *p* < 0.0001. NS means not significant.

The regulation of cell motility is ultimately achieved by influencing its own motility components, including flagellum [26], chemotaxis [26], and membrane potential [24]. Together with the previously published studies reporting the role of amino acids in bacterial motility [27], we speculated that serine might affect these motility components. To verify this, serine was added to swimming medium at the indicated concentrations and the expression of flagella and chemotactic genes was measured by qRT-PCR (Figure 7A). As shown in Figure 7B, the addition of 40 mM serine suppressed almost all flagella-related genes except for *motA* and *motB* encoding flagellar motor protein, implying that serine did not regulate flagella by affecting flagellar motor protein pathways to influence bacterial motility. Among these serine-inhibited genes, *fliM*, *flhB*, and *fliC* were most significantly repressed, which encodes flagellar motor switch protein, flagellar biosynthesis protein, and flagellin, respectively. Similarly, addition of serine inhibited most of the chemotaxis-related genes in *H. alvei* H4 (Figure 7C). Interestingly, low concentrations of serine promoted *cheZ* expression, while high concentrations of serine inhibited it. In particular, the expression of *cheR* was markedly inhibited by serine. The above results indicated that serine may affect bacterial chemotaxis through the chemotaxis protein-glutamate O-methyltransferase pathway, thereby interfering with bacterial motility. Overall, it was suggested that QS-regulated serine was capable of repressing most flagella/chemotaxis-related genes, and these results may partially explain why serine can inhibit bacterial motility.

To determine the effect of QS-regulated serine on bacterial membrane potential, cells were collected from swimming agar medium supplemented with indicated concentrations of serine, and the membrane potential of the cells was measured by using the dye thioflavin T (ThT, Figure 7D). According to a previously published study [24], accumulation of this fluorescent dye inside the cells and thus the fluorescent density of the cells anti-correlate with the membrane potential of the cells. As shown in Figure 7E, the majority of cells collected from the swimming agar medium without adding serine exhibited intense fluorescence intensity (shown as cyan fluorescent protein (CFP)), indicating a low membrane potential of the cells. In contrast, the majority of those cells collected from the swimming medium supplemented with the indicated concentrations of serine exhibited very weak fluorescence, and the intensity of fluorescence decreased with increasing serine concentrations, indicating high membrane potentials of the cells. Furthermore, cells were collected from the periphery and center of colony biofilms with and without serine addition and it was found that the membrane potential at the periphery of the colony was lower than that at the center of the colony biofilm. In addition, the expression of selected genes (*sdhC*, *gpsA*, *ndhF*, *sdhB* and *menH*), encoding for enzymes involved in electron transfer was investigated by qRT-PCR from three different swimming media (adding 0, 5 mM, and 40 mM serine). Interestingly, low concentrations of serine promoted *ndhF* expression, while high concentrations of serine inhibited its expression. Compared to the 0- and 5 mM groups, 40 mM serine promoted high expression of *sdhC*, *gpsA*, *sdhB* and *menH* (Figure 7F). Finally, the effect of QS-regulated serine on key genes of the QS system (*luxI* and *luxR*, encoding signal synthesis and receptor, respectively) was investigated, and it was found that addition of different concentrations of serine could promote the expression of these QS genes (Figure 7G). However, it is not clear whether that positive feedback loop between serine and the QS genes is necessary to induce all the phenotypic changes observed with the addition of serine. The phenotypes of swimming and Congo red were selected to compared the phenotypic differences between *H. alvei* WT and Δ*luxI* under the conditions of adding 5 mM and 40 mM serine (Supplementary Figure S3). For the swimming phenotype, adding 40 mM serine significantly inhibited the swimming ability of both strains. Further comparison revealed that the colonies of WT strains exhibited a more even and dense colony morphology, while Δ*luxI* only formed a thicker colony morphology at the sampling points. For the Congo red phenotype, there were significant differences between the phenotypes of both strains, regardless of whether low or high concentrations of serine were added. In summary, these results suggested that QS-regulated serine had a strong effect on the expression of cell motility-related components, and can also feedback activate the QS system to further enhance the comprehensive regulation ability of *H. alvei* H4.

## 4. Discussion

**Figure 8:**
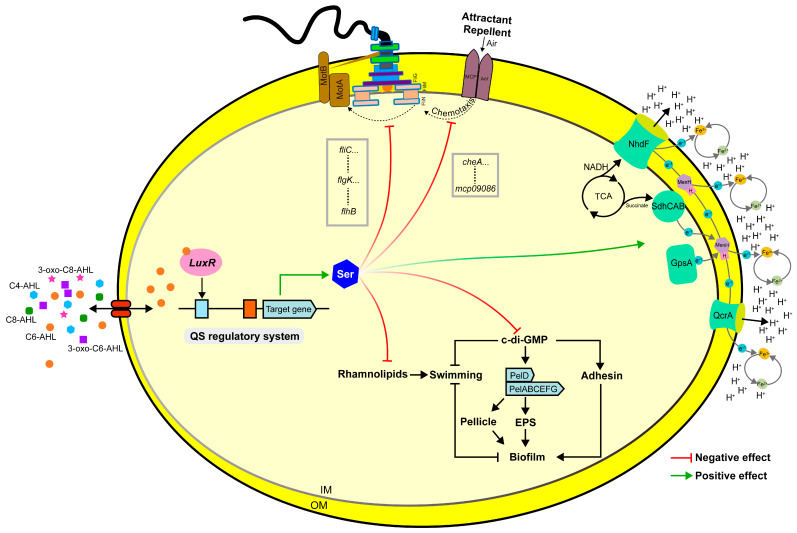
Regulation model of QS-regulated serine affecting bacterial motility.

The debate over the relative contributions of genetic determinants versus environmental conditions to bacterial phenotypes has been longstanding in microbiology [14]. In the context of QS and its influence on spoilage phenotypes, most studies, including our prior work, have focused on the phenotypic consequences of QS gene deletion or signal molecule add-back, establishing the global regulatory role of the AHL-type QS system in biofilm formation and motility [15,28]. However, the mechanistic understanding of how QS-induced metabolic differences translate into phenotypic outcomes remains limited. This study aimed to address this gap by investigating the QS system of the foodborne spoilage bacterium *H. alvei* H4. Based on our results, we constructed a regulatory network wherein QS-promoted serine accumulation inhibits bacterial motility by affecting multiple motility-related factors, including rhamnolipids, c-di-GMP, flagella, chemotaxis, and membrane potential (Figure 8).

The role of serine in bacterial physiology extends beyond its function as a proteinogenic amino acid, serving as a key node in one-carbon metabolism, nucleotide synthesis, and phospholipid biosynthesis. Its influence on bacterial behavior has been documented in diverse contexts. For instance, serine starvation is known to trigger stringent-response-like pathways and biofilm induction through translational pausing at serine codons, leading to decreased expression of motility repressors such as *SinR* in *Bacillus subtilis* [29]. Conversely, exogenous serine supplementation has been shown to modulate flagellar function: in *Vibrio* species, serine directly binds to the *PomB* stator subunit, allosterically inhibiting motor torque generation without affecting flagellar assembly or chemotactic signaling [30]. In *Staphylococcus* spp., D-serine interferes with cell-wall adhesion machinery, reducing biofilm attachment independently of growth inhibition [31]. Here, the QS-promoted accumulation of extracellular serine leads to concerted downregulation of flagellar/chemotaxis operons and suppression of rhamnolipid biosynthesis genes. Indeed, serine functions as a QS-dependent metabolic effector that influenced gene expression. This transcriptional reprogramming suggests that serine operates as an inter-cellular metabolic cue within the QS network, shifting population behavior from a motile, exploratory state to a more sessile, aggregated lifestyle—a strategy that may enhance survival in structured environments such as food surfaces. These results indicated serine has been evolutionarily co-opted into different regulatory niches across bacteria.

A central and novel finding of this work is the identification of serine as a key upstream regulator of rhamnolipid metabolism and its interplay with the c-di-GMP network in *H. alvei* H4. Rhamnolipids are well-characterized biosurfactants in bacteria like *Pseudomonas aeruginosa*, where they facilitate surface motility, biofilm structuring, and virulence [18,25]. This study found that serine at concentrations as low as 1.25 mM effectively suppresses the production of rhamnolipid, indicating that serine may inhibit the motility ability of bacteria by suppressing rhamnolipid [17]. Furthermore, the QS system of *H. alvei* H4 itself can promote the accumulation of rhamnolipids, suggesting that the complementary QS regulatory mode enhanced the adaptability of the cell to the complex and changeable external environment. However, some studies have also reported the inhibition of QS on the rhamnolipid synthesis genes [32], which may not be a direct regulation of QS systems, but a cascade regulation caused by QS changes in the metabolic environment. Our results further reveal that serine’s regulatory influence extends to the second messenger c-di-GMP. C-di-GMP is a master regulator of the motile-to-sessile transition, with high levels generally promoting biofilm formation and repressing motility [17]. Intriguingly, we observed that a high concentration of serine (40 mM) significantly reduced intracellular c-di-GMP and modulated the expression of PDE and DGC genes (Figure 6F,G). The co-occurrence of rhamnolipid suppression and c-di-GMP reduction presents a seemingly paradoxical scenario, as the latter is typically associated with a promoted motile state. We propose a model to reconcile these observations: serine exerts its dominant inhibitory effect on motility by strongly repressing the rhamnolipid-dependent “execution pathway” essential for surface translocation, an effect so potent that it overrides any potential pro-motility signal arising from lowered c-di-GMP. The modulation of the c-di-GMP network by serine might therefore serve as a fine-tuning or compensatory mechanism.

Flagellar assembly and chemotactic signaling constitute the core execution modules for bacterial motility, integrating mechanical propulsion with environmental sensing [33,34]. In *H. alvei* H4, our findings demonstrate that serine functions as a potent transcriptional repressor of this entire motility apparatus. The significant downregulation of flagellar genes such as *fliM* (encoding the motor switch protein) and chemotaxis genes including *cheR* (encoding the adaptation methyltransferase) provides direct mechanistic evidence for the observed motility inhibition. This transcriptional repression is particularly noteworthy because it targets genes involved in both motor function (*fliM*) and sensory adaptation (*cheR*), suggesting serine exerts coordinated control over motility at multiple regulatory levels. Interestingly, this serine-mediated repression aligns with previous observations of QS-motility connections (Figure 1D). While Mellbye et al. reported that QS downregulates flagellar genes in *Nitrobacter winogradskyi* [35], and Silagyi et al. showed AI-2-mediated QS regulates chemotaxis and motility genes in *Escherichia coli* [36], these studies did not identify the specific metabolic mediators involved. Our work bridges this gap by identifying serine as the key effector molecule executing this QS-mediated repression. The connection becomes particularly significant when considering the work of Afanzar et al. and Burkart et al., which established serine’s role in flagellar and chemotaxis regulation through different mechanisms [33,37]. What emerges from integrating these findings is a coherent regulatory paradigm: the QS system utilized serine as a metabolic signal to synchronize motility behavior with population density in *H. alvei* H4. At high cell density, QS promoted serine accumulation, which then acts as both an intracellular signal and potential intercellular cue to downregulate the flagellar/chemotaxis regulon. This represents a “metabolic override” strategy where amino acid metabolism directly reprograms gene expression patterns of motility.

Traditionally, the importance of membrane potential has been discussed in the context of molecule transport, ATP generation, biofilms, and cell division [24]. In our study, a link between membrane potential and QS-regulated serine was demonstrated, and it was showed that serine enhanced the membrane potential mainly by regulating the expression of *gpsA* and *menH* encoding for enzymes involved in electron transfer [24]. This was consistent with the research of Reitz et al., which also demonstrated that serine can facilitate the acquisition of ions by participating in the synthesis and evolution of siderophores-endothelin [38]. Previous studies showed that the influence of ions on bacterial motility is complex. For example, Takekawa et al. reported that flagella driven by ions can promote the motility of bacteria [39], while Banin et al. found that *Pseudomonas aeruginosa* exhibited incessant twitching motility on surfaces at low iron concentrations, but the mechanism for iron control of twisting mobility is unknown [40]. The reasons for this may be that membrane potential plays an important role in regulating the transport of some important metabolites such as glutamate, which in turn can affect bacterial motility and biofilms formation [24]. This suggested that the microbial regulation system participates in more physiological activities by regulating some functional metabolites, which is a universal regulation mode in physiological response.

## 5. Conclusions

In summary, this study elucidated that QS system in *H. alvei* H4 regulates spoilage-related phenotypes through the modulation of amino acid metabolism, with serine identified as a key metabolic effector. By integrating metabolomic profiling with phenotypic and transcriptomic analyses, we delineated a multi-tiered regulatory network: the QS system promotes extracellular serine accumulation, which in turn inhibits bacterial motility by suppressing rhamnolipid biosynthesis, downregulating flagellar and chemotaxis gene expression, modulating membrane potential, and influencing c-di-GMP signaling. These coordinated effects collectively drive a transition from a motile to a sessile lifestyle, thereby also impacting biofilm formation. It reveals that bacterial behavioral shifts are not solely directed by signaling molecules but are also orchestrated through defined metabolic reprogramming. The identification of serine as a functional nexus between QS and motility provides a novel target for potential intervention strategies aimed at controlling biofilm formation and spoilage in complex food systems. Future studies may focus on elucidating the direct molecular targets of serine and validating this regulatory axis in real food matrices to facilitate the development of metabolite-based food preservation approaches.

## Data Availability

The data supporting the findings of this study, including raw images and mass spectrometry files, were available upon request from the corresponding author.

## Supplementary Materials

The following supporting information can be downloaded at: https://www.mdpi.com/article/10.3390/foods15020281/s1, Figure S1. The 200-permutation test to validate the OPLS-DA model. Figure S2. Effect of different concentrations of serine on the biofilm formation of H. alvei H4. Figure S3. Phenotypic comparison between H. alvei WT and ΔluxI under the condition of adding different concentrations of serine, including swimming phenotype (A) and Congo red phenotype (B).
