# Supplementary material for: The Regulatory Role of Quorum Sensing-Mediated Amino Acid Metabolism in Biofilm Formation and Motility of Hafnia alvei H4
Source: Foods. 2026 Jan 12;15(2):281. doi: 10.3390/foods15020281 (PMC12841376; doi:10.3390/foods15020281)
Supplement: Supplementary file 1 [file foods-15-00281-s001.zip › foods-4058478-supplementary.pdf]

Supplementary Information

Journal name: *foods*

Manuscript Title: The regulatory role of quorum sensing-mediated amino acid metabolism on biofilm formation and motility of *Hafnia alvei* H4

The name(s) of the author(s): Congyang Yan[1,2,3], Xue Li[1,2], Gongliang Zhang[1,2], Jingran Bi[1,2], Hongshun Hao[2], Hongman Hou[1,2*]

The affiliation(s) and address(es) of the author(s):

[1]School of Food Science and Technology, Dalian Polytechnic University, Dalian, China

[2]Liaoning Key Lab for Aquatic Processing Quality and Safety, Dalian, China

[3]College of Grain Science and Technology, Shenyang Normal University, Shenyang, China

Address: Liaoning Key Lab for Aquatic Processing Quality and Safety, No. 1, Qinggongyuan, Ganjingzi District, Dalian, Liaoning 116034, People's Republic of China.

*** Correspondence:**

Hongman Hou

Email: houhongman@dlpu.edu.cn

Tel.: +86-411-8632-2020

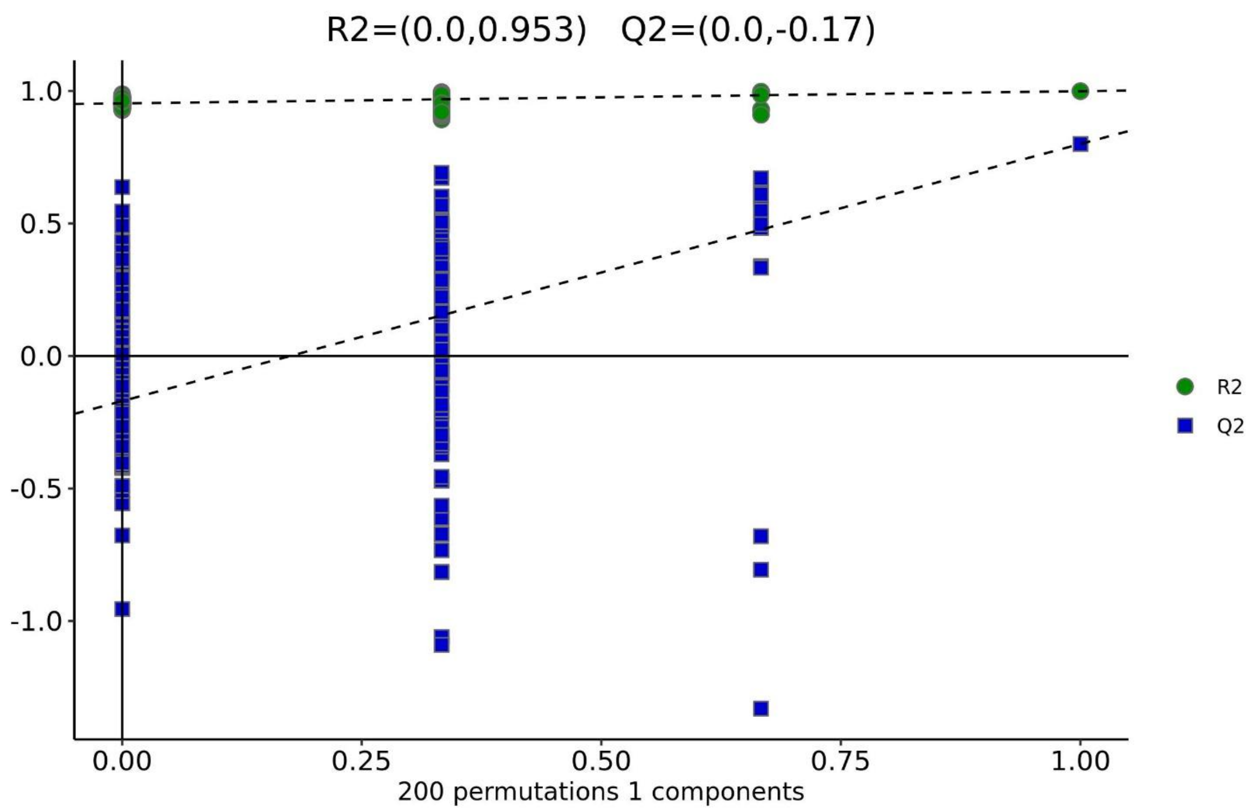

Figure S1. The 200-permutation test to validate the OPLS-DA model.

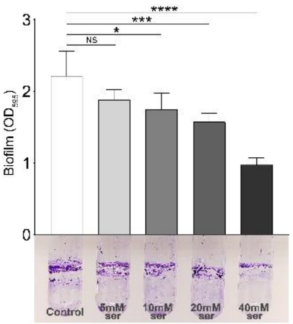

Figure S2. Effect of different concentrations of serine on the biofilm formation of *H. alvei* H4.

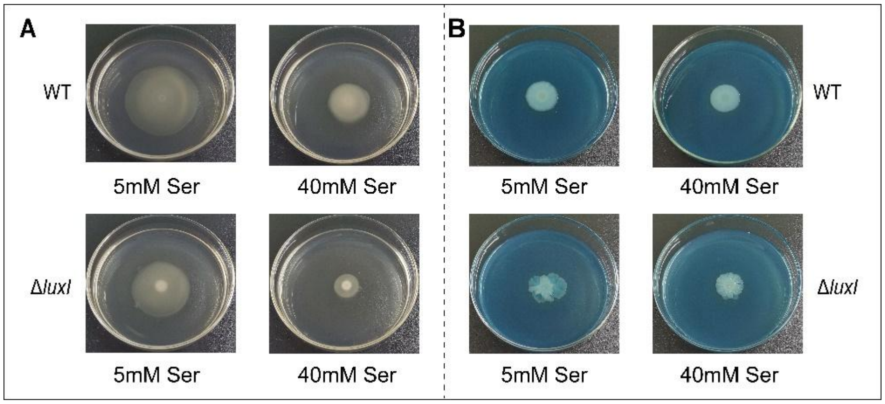

Figure S3. Phenotypic comparison between *H. alvei* WT and Δ*luxI* under the condition of adding different concentrations of serine, including swimming phenotype (A) and Congo red phenotype (B).
